# Identification of sulfur components enhancing the anti-*Candida* effect of *Lactobacillus rhamnosus* Lcr35

**DOI:** 10.1038/s41598-020-74027-7

**Published:** 2020-10-13

**Authors:** Caroline Dausset, Stéphanie Bornes, Sylvie Miquel, Nathalie Kondjoyan, Magaly Angenieux, Laurence Nakusi, Philippe Veisseire, Elina Alaterre, Luis G. Bermúdez-Humarán, Philippe Langella, Erwan Engel, Christiane Forestier, Adrien Nivoliez

**Affiliations:** 1 Research and Development Department, BIOSE, 24 avenue Georges Pompidou, 15000 Aurillac, France; 2 Micalis Institute, INRAE, AgroParisTech, Université Paris-Saclay, 78350 Jouy-en-Josas, France; 3 Université Clermont Auvergne, CNRS, Laboratoire Microorganismes : Génome et Environnement, 63000 Clermont-Ferrand, France; 4 Université Clermont Auvergne, INRAE, VetAgro Sup, UMRF, 15000 Aurillac, France; 5 INRAE, UR370 QuaPA, Microcontaminants, Aroma & Separation Science Group (MASS), 63123 Saint-Genès-Champanelle, France; 6 Present Address: HORIBA ABX SAS, Parc Euromédecine, Rue du Caducée, BP 7290, 34184 Montpellier Cedex 4, France

**Keywords:** Antimicrobials, Bacteria, Fungi

## Abstract

GYNOPHILUS (Lcr REGENERANS) is a live biotherapeutic product (LBP) aimed at restoring the vaginal microbiome and contains the live biotherapeutic microorganism *Lactobacillus rhamnosus* Lcr35. In this study, the LBP formulation and manufacturing process significantly enhanced the anti-*Candida* activity of *L. rhamnosus* Lcr35, with a complete loss of viability of the yeast after 48 h of coincubation. Sodium thiosulfate (STS), one excipient of the product, was used as a potentiator of the anti-*Candida* spp. activity of *Lactobacilli*. This contact-independent phenomenon induced fungal cell disturbances, as observed by electron microscopy observations. Nonverbal sensory experiments showed clear odor dissimilarities between cocultures of *L. rhamnosus* Lcr35 and *C. albicans* in the presence and absence of STS, suggesting an impact of odor-active metabolites. A volatolomic approach allowed the identification of six odor-active compounds, including one sulfur compound that was identified as S-methyl thioacetate (MTA). MTA was associated with the antifungal effect of Lcr35, and its functional link was established in vitro. We show for the first time that the LBP GYNOPHILUS, which is a highly active product in the reduction of vulvovaginal candidiasis, requires the presence of a sulfur compound to fully achieve its antifungal effect.

## Introduction

Vulvovaginal candidiasis (VVC) is the most common vaginal pathology associated with the presence of *Candida* spp. and it affects 70–75% of women at least once in their live^1,2^. This disorder has a very negative impact on a woman’s quality of life and represents significant healthcare costs. Antifungal treatments fail to restore the protective microbiota and to prevent against VVC symptoms. Conventional therapies are often associated with side-effects such as risk increase for bacterial vaginosis (BV), severe disturbances on vaginal microbiota, and recurrence^3,4^. The relapse rate, as defined by a diagnosed symptomatic infection within 6 months post oral or vaginal antifungal treatment, is high (60–70%), leading to important incidence of recurrent VVC (defined by the occurrence of at least 4 VVC episodes per year), estimated at 5–10%^5–7^. A new therapeutic approach is the use of live beneficial microorganisms to restore the local microbiota balance.

Live biotherapeutic products (LBP) are defined by U.S. Food and Drug Administration (FDA) as “live microorganisms (i.e., bacteria, yeast) with an intended therapeutic or preventive effect in humans, regardless of the route of administration” except vaccines^8–10^. LBPs contain one or more live biotherapeutic microorganisms (LBM). GYNOPHILUS (Lcr REGENERANS) LBP contains *Lactobacillus rhamnosus* Lcr35, a LBM whose in vitro and in vivo characteristics are well-documented^11–15^. This LBP is recommended in preventive and curative therapies for gynecological indications, mostly vulvovaginal candidiasis^16–18^.

Clinical efficiency of the use of LBP GYNOPHILUS in the prevention of recurrent VVC and BV has been shown^19–21^. GYNOPHILUS supply increased the clinical and microbiological efficacy of the antimicrobial treatment and promote significantly the resilience of the vaginal microbiota^17,19–21^. In vitro*,* the LBM Lcr35 and its galenic vaginal forms, LBP GYNOPHILUS, were shown to impair the growth of *C. albicans* and to induce a fungicidal effect after 24 h of co-incubation^22^.

In addition to the bacterial strains, the galenic forms contain nutrients, cryoprotectants and flow agents that are necessary to bacterial stability along the manufacturing process and for viability conservation. LBP formulation and production parameters have already been shown to promote the LBM properties, notably the anti-*Candida* effect of the Lcr35 in GYNOPHILUS^22^. Among the different molecules added during the manufacturing process, sulfur compounds have been widely used and sodium thiosulfate (STS) is part of the excipients of GYNOPHILUS. The aim of this study was to assess the impact of STS on the anti-*Candida* properties of the LBM Lcr35. Determination of the viability of *C. albicans* in co-incubation assays with Lcr35 indicated that STS enhanced its antifungal activities. This effect was associated with the presence of volatile organic compounds (VOCs) and we were able to identify one sulfur containing active component, S-methyl thioacetate, using a volatolomic approach.

## Results

### Antifungal of Lcr35 potentiated by STS

**Figure 1:**
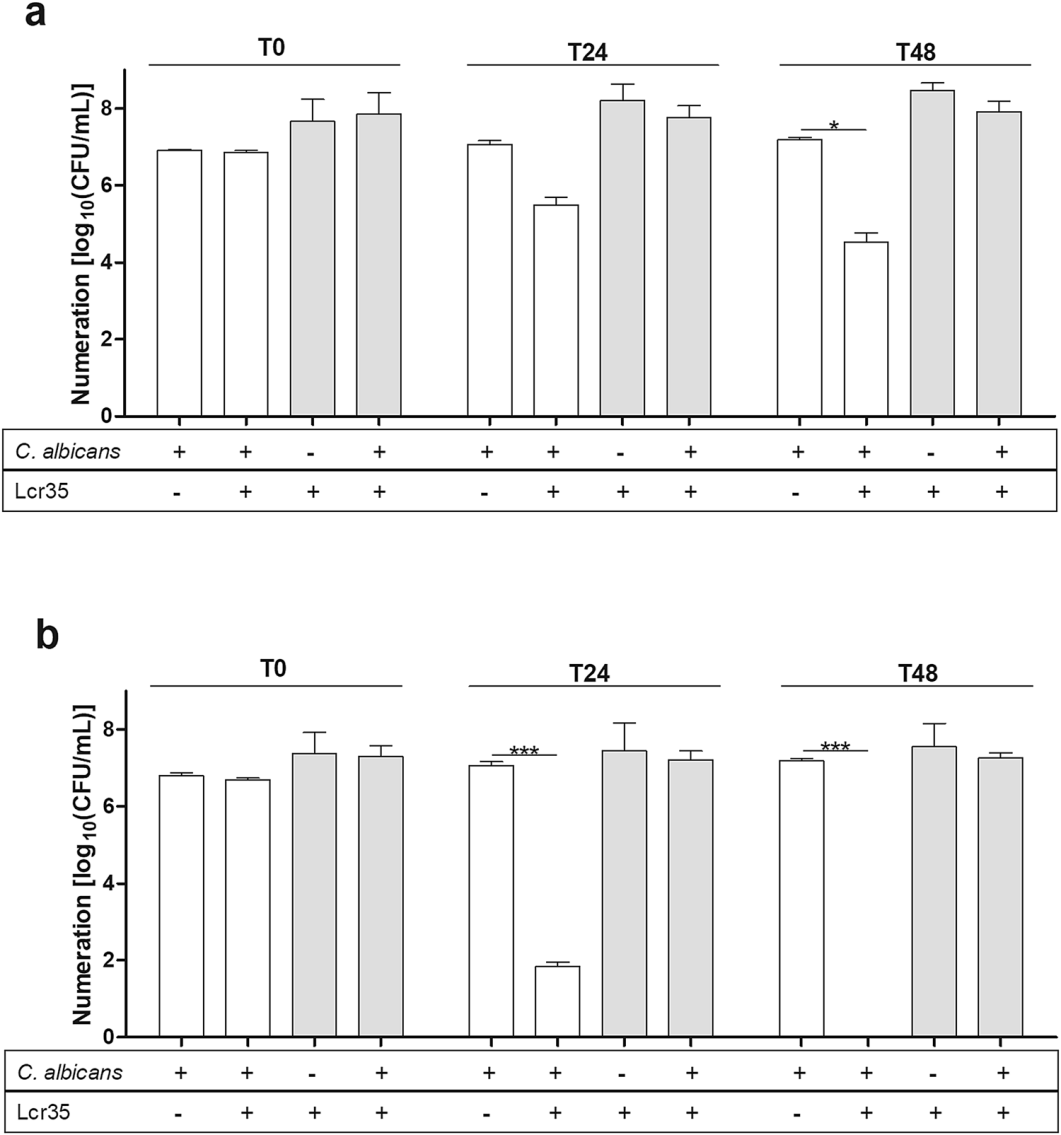
Effect of Lcr35 on the viability of *Candida albicans* ATCC 10231 in the absence (**a**) and in the presence (**b**) of STS (1 g/l). Viability of *C. albicans* white bar and Lcr35 grey bar determined at strain contacting (T0), after 24 h (T24) and 48 h (T48) of co-incubation with Lcr35 (**a**), and with STS (1 g/l) (**b**). Controls were incubated in same conditions. N = 3, *p < 0.05, **p < 0.01, ***p < 0.001 (one-way ANOVA test and Bonferroni correction).

*C. albicans* ATCC 10231 viability was reduced by 2.7 log_10_ (CFU/ml) after 48 h as compared to the non-treated control (Fig. 1) without any impact onto Lcr35 viability (Fig. 1a). With STS supplementation, Lcr35 induced a 5.2 log_10_ (CFU/ml) decrease of *C. albicans* viability after 24 h and a complete loss of viability after 48 h of co-incubation (Fig. 1b). A kinetic study performed by plate counting and cytometry combined with propidium iodide showed that *C. albicans* viability was affected as early as after 6 h of co-incubation (Fig. S1a,b). However, STS alone did not affect *C. albicans* growth compared to the control condition (Fig. 1a,b). Similarly, STS supplementation at 1 g/l did not affect Lcr35 viability in control nor in co-incubation conditions. At higher concentrations, the viability of Lcr35 was impaired (Fig. S2a) but no effect was observed with *C. albicans* (Fig. S2b). In addition, eight other *Lactobacillus* spp. were tested for their anti-*Candida* effect in the same conditions: all of them but *L. reuteri* induced a 7 log_10_ decrease of viability of the pathogen compared to the control after 48 h of co-incubation (Fig. S3).

**Figure 2:**
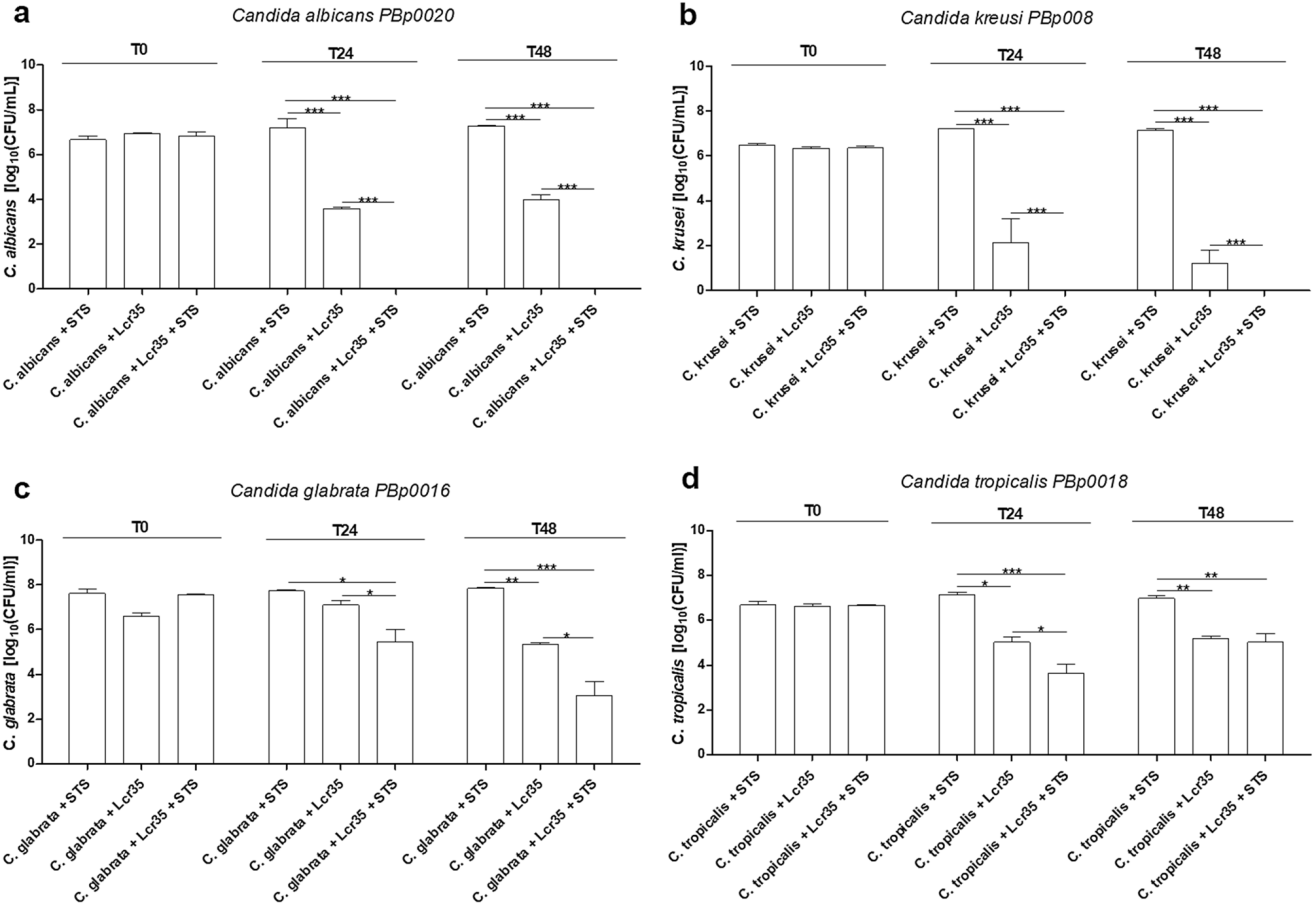
Viability of four clinical *Candida* spp co-incubated with Lcr35 with and without STS (1 g/l). *C. albicans* (**a**), *C. krusei* (**b**), *C. glabrata* (**c**), *C. tropicalis* (**d**) viability determined at strain contacting (T0), after 24 h (T24) and 48 h (T48) of co-incubation with Lcr35 with or without 1 g/l of STS. *Candida* spp. controls were incubated without Lcr35 and with 1 g/l STS. N = 3, *p < 0.05, **p < 0.01, ***p < 0.001 (one-way ANOVA test and Bonferroni correction).

The antifungal effect of Lcr35 was also efficient on other four *Candida* spp. (Fig. 2). During co-incubation with Lcr35, *C. albicans* clinical isolates viability was reduced by 3.3 log_10_ (CFU/ml) as observed on *C. albicans* reference (ATCC 10231) (Fig. 2a). When Lcr35 culture medium was supplemented with STS, no viable cells of *C. albicans* and *C. krusei* clinical strains were detected after 24 h of co-incubation. *C. glabrata* and *C. tropicalis* viability were respectively reduced by 2.5 log_10_ (CFU/ml) and 3.5 log_10_ (CFU/ml) at the same time (Fig. 2c,d). After 48 h of co-incubation with Lcr35 and STS, *C. glabrata* growth was inhibited to 4.8 log_10_ (CFU/ml) (Fig. 2c). Moreover, after 48 h of co-incubation with Lcr35 and STS, no viable cells of six other clinical *C. albicans* were detected (Fig. S4), similarly to *C. albicans* reference strain (ATCC 10231) (Fig. 1).

### Lcr35 alters the morphological structure of *C. albicans* in the presence of STS

**Figure 3:**
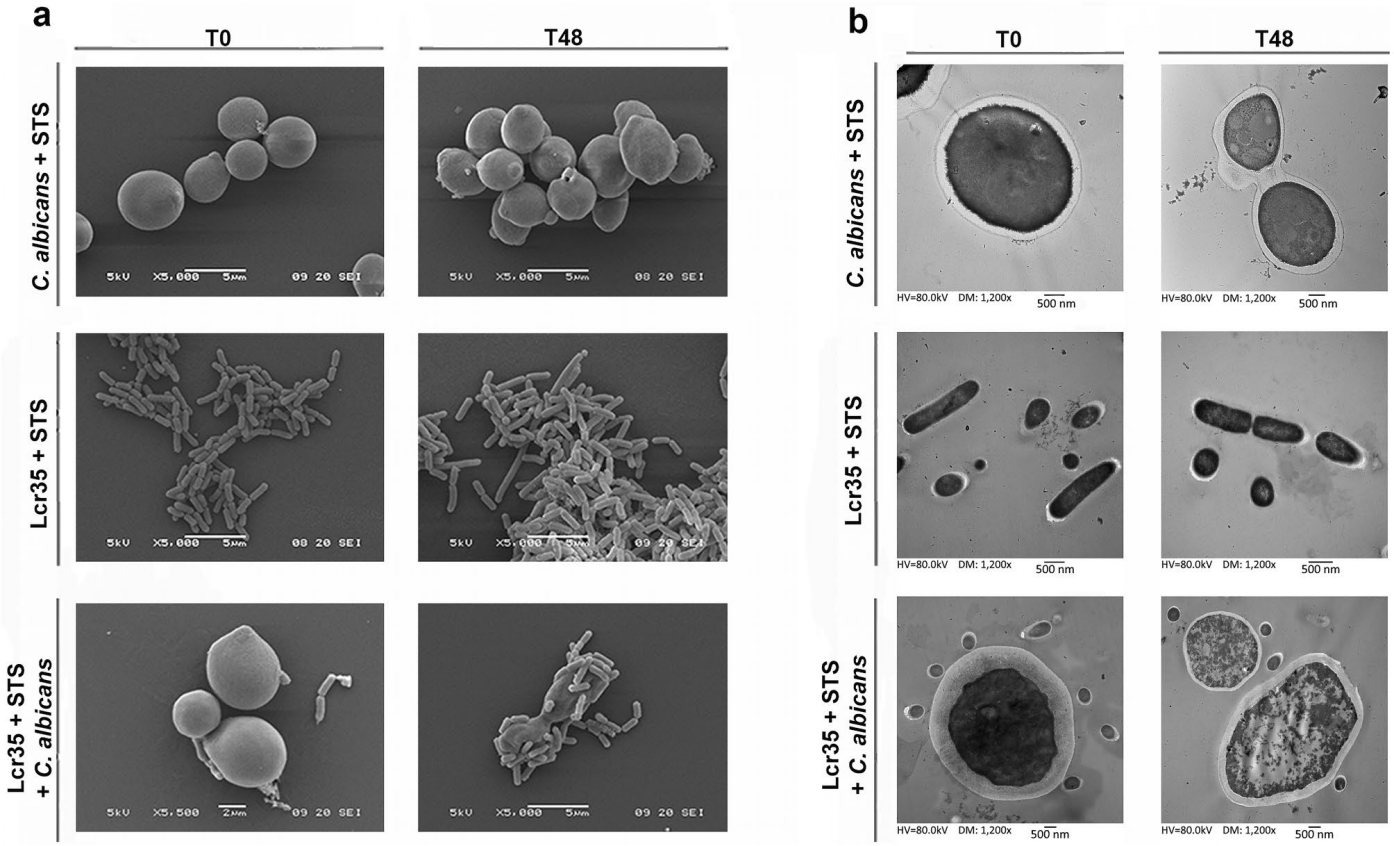
Scanning electron microscopy and transmission electron microscopy observations of *Candida* and Lcr35 at strain contacting (T0) and after 48 h of co-incubation (T48). SEM (**a**) and TEM observations (**b**) of *C. albicans* and Lcr35 co-incubated with STS (1 g/l). As a control, microorganisms were incubated with STS containing MRS broth.

Scanning Electron Microscopy (SEM) observations showed that *C. albicans* cells lost their homogeneous and regular surface after 48 h of co-incubation with Lcr35 and STS, without affecting the Lcr35 apparent morphology (Fig. 3). Similar effect was observed when *C. albicans* was incubated with Lcr35 in the absence of STS (data not shown). Neither Lcr35 nor *C. albicans* cellular integrity were impacted by STS supplementation in pure culture (Fig. 3a,b). Transmission Electron Microscopy (TEM) observations showed important alterations of yeast intracellular integrity after incubation in presence of Lcr35 and STS, contrary to control observations performed in the absence of Lcr35 and STS (Fig. 3b).

### Contact-independent anti-*Candida* activity of Lcr35

**Figure 4:**
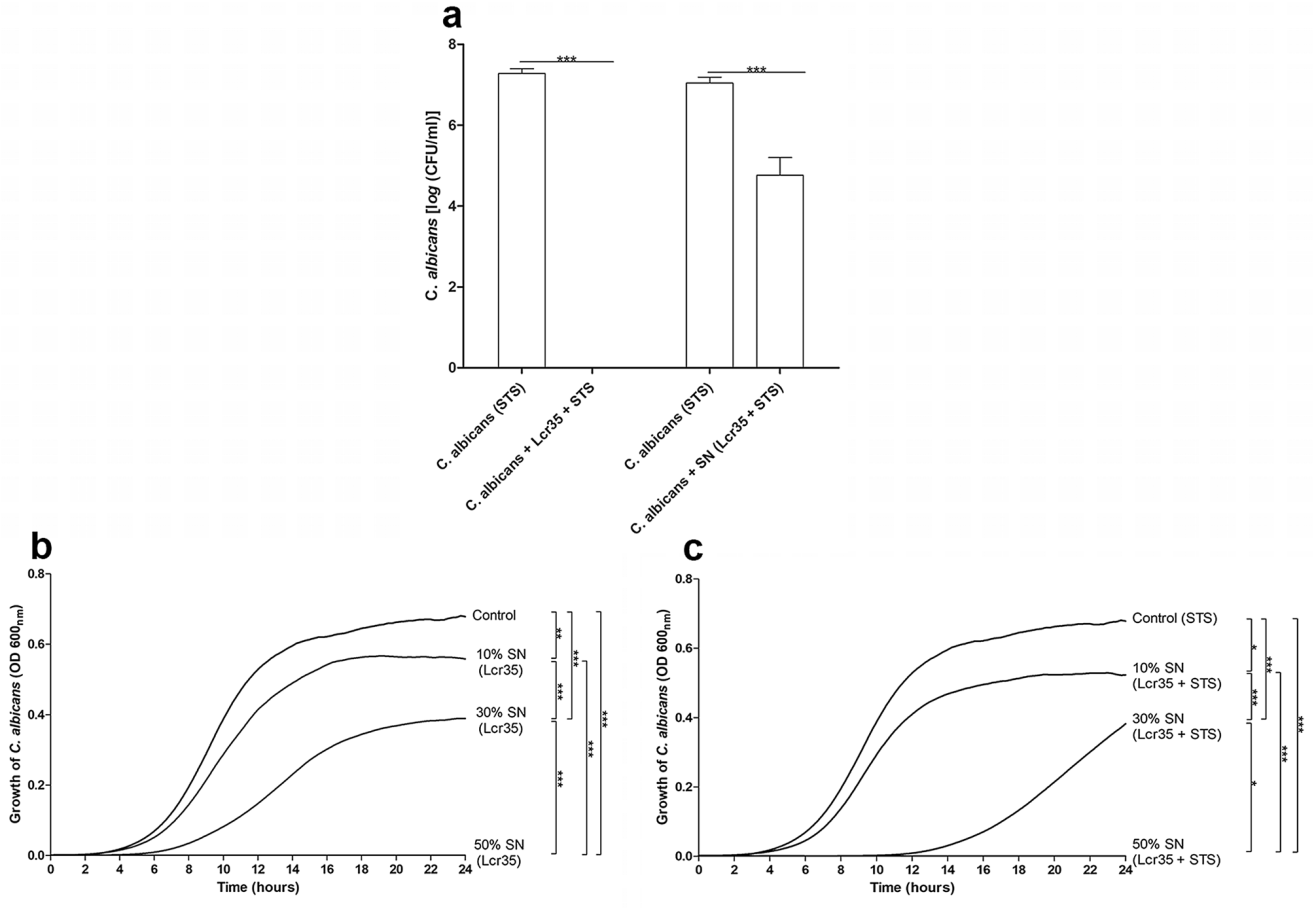
The impairment of viability and growth of *C. albicans* by Lcr35 is contact independent. The proximity-dependent mechanism of Lcr35 was tested using cell culture inserts (**a**). The microorganisms were cultivated with 0.4 µm-pore-size membrane to separate cells with Lcr35 in the bottom chamber and *Candida albicans* in the top chamber. Anti-*Candida* effect of Lcr35 cell free supernatant was evaluated on *C. albicans* viability at 37 °C over 48 h (**a**). The impact of cell free supernatant on *C. albicans* growth was evaluated during 24 h after inoculation in medium with Lcr35 supernatant (**b**) or Lcr35 + STS cell free supernatant (**c**). The *C. albicans* growth was followed by OD_600 nm_ every 15 min. *C. albicans* control was inoculated in MRS (**b**) or MRS with 1 g/l STS (**c**). N = 3, *p < 0.05, **p < 0.01, ***p < 0.001 (one-way ANOVA test and Bonferroni correction).

To further characterize anti-*Candida* mechanisms, contact-dependent inhibition assays were performed using cell culture insert. An equivalent antifungal activity of Lcr35 and STS onto *C. albicans* was observed when cells and bacteria were directly in contact or separated by a cell culture insert (Fig. 4a). However, the culture cell free supernatant induced only a loss of 2 log_10_ of *Candida albicans* viability (Fig. 4a).

The influence of cell free supernatants from 48 h-old Lcr35 culture with and without STS was also evaluated on *C. albicans* growth (Fig. 4b,c). Whatever the concentration of the cell free supernatant tested (10%, 30% and 50%), a significant inhibition of *C. albicans* growth was observed compared to the control condition performed using 50% of sterile water (p < 0.01) (Fig. 4b). *C. albicans* growth was inhibited by the presence of 10% and 30% of Lcr35 supernatant, with or without STS (Fig. 4c). In culture condition containing 30% of Lcr35 + STS supernatant, *C. albicans* growth was delayed for 8 h compared to Lcr35 supernatant (30%). Using 50% supernatant, no *C. albicans* growth was observed.

Additionally, protease and heat (100 °C for 15 min) treatments had no effect on the fungistatic activity of the Lcr35 cell free supernatant. Using ultrafiltration, only the fraction including molecular weight smaller than 10 kDa had an inhibition effect of *C. albicans* growth (data not shown).

### Identification of the volatile compounds involved in anti-*Candida* activity of Lcr35

**Figure 5:**
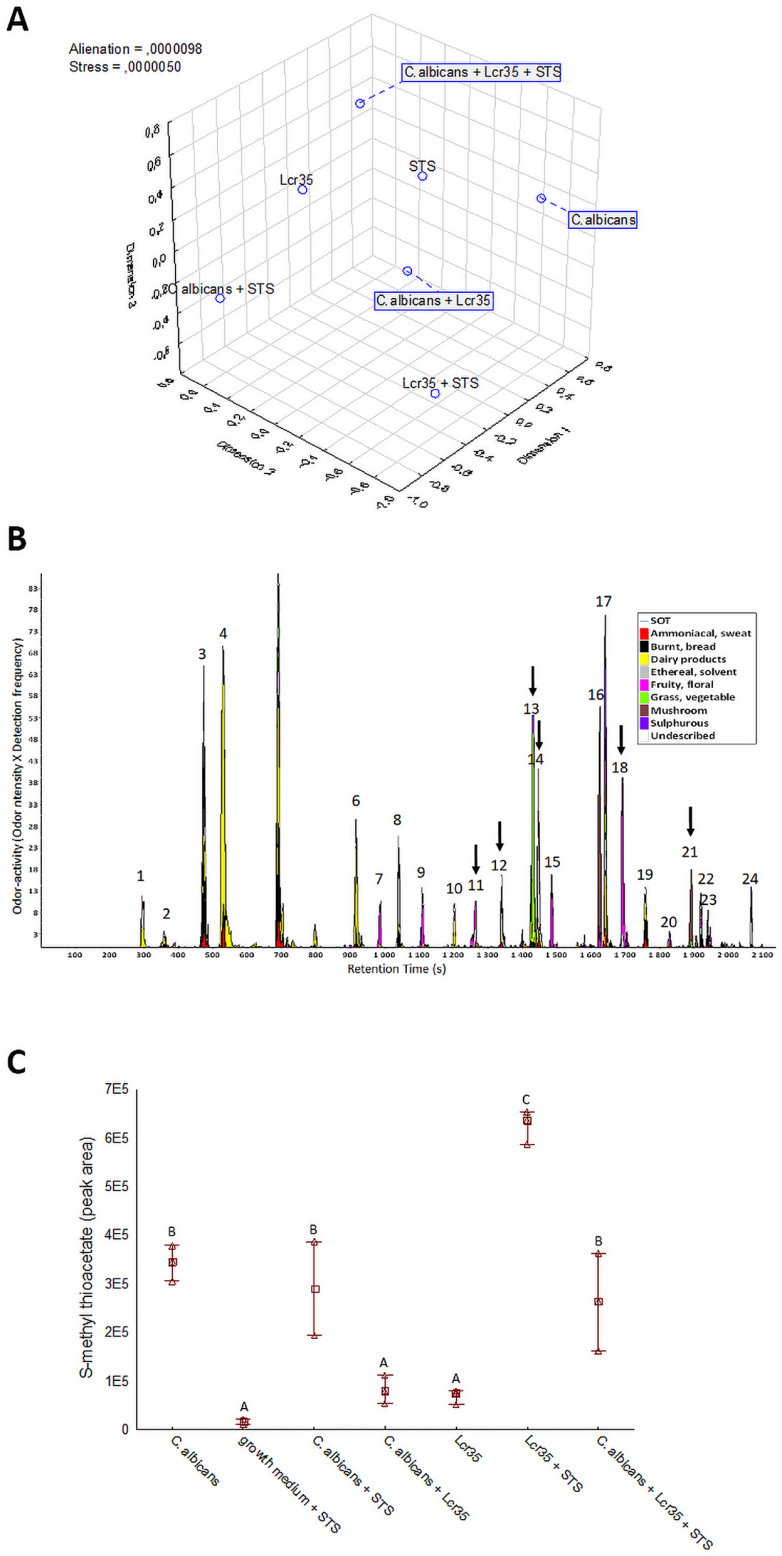
Odor dissimilarity-testing and volatolomic approach indicated the involvement of S-methyl thioacetate in the Lcr35 anti-*Candida* effect. Odor dissimilarities of seven conditions corresponding to culture media inoculated with *C. albicans*, Lcr35 and STS, alone or in combinations were assessed by non-verbal sensory evaluation thanks to 2D mapping (n = 25 assessors). Distance matrices were compiled and treated by MDS to determine the 3D representation of the odor dissimilarities (**a**). Odor-active compounds of the average of four conditions (Lcr35, *C. albicans* + Lcr35, Lcr35 + STS and *C. albicans* + Lcr35 + STS) were pooled in the aromagram profile (**b**). The peak numbers refer to odor-active compounds given in Table S4 (supporting information). Dark arrows indicate significant odor-active markers according to Kruskal–Wallis non-parametric test (p < 0.05). Abundance in S-methyl thioacetate (**c**) extracted after 12 h of incubation by GC–MS in the 7 conditions. Tukey multiple mean comparison test (p < 0.05) was performed on DH-GC–MS peak abundances—Means with the same letters (a–c) are not significantly different.

The 25 panelists non-verbal sensory experiment had determined the odor dissimilarities between seven conditions analyzed after 12 h of incubation (Fig. 5a). The map (2,3) showed a clear difference between the odors of *C. albicans* culture, *C. albicans* and Lcr35 co-culture and *C. albicans* co-incubated with Lcr35 and STS, suggesting a different modulating impact of Lcr35, Lcr35 with STS and *C. albicans* on odor-active metabolites produced*.* In addition, the 3D representation confirmed that STS modulates the odor of pure cultures of *C. albicans* and Lcr35. The odor of this binary combinations (*C. albicans* and STS, Lcr35 and STS) differed from the odor of the control medium inoculated with STS alone (STS) confirming that the odor of STS excipients may not explain the odor differences. The volatolomic analysis of seven conditions of *C. albicans* and Lcr35 cultures revealed different volatile profiles. Figure 5b presents the average aromagram obtained by compiling the aromagrams of the four samples points out 24 odor zones. Out of them, 15 odor zones were classified in one of the dairy (1–5, 6, 10), fruity floral (7, 9, 11, 15, 18), grass-vegetable (13) or mushroom (16, 21) odor poles whereas the 9 remaining ones were less consensually described by the panelists. Cross analyses of these odor zones with significant MS data at corresponding retention time enabled the tentative identification of most odor-active compounds (Fig. S5). Six out of these tentatively identified odor-active compounds were found to discriminate the four cultures according to their odor-activity, confirming the existence odor-active markers of the four cultures investigated.

Among them, one sulfur compound was identified the 3-methylthiopropanal. Then to extend the marker discovery to VOC, a focus on sulfur VOCs determined four discriminate sulfur compounds including *S*-methyl thioacetate, dimethyldisulfide, 3-methylthiopropanal and 2-acetylthiazole. Only the *S*-methyl thioacetate (MTA, CAS 1534-08-3) was discriminating between the conditions analyzed (Fig. 5c). Production of MTA by *C. albicans* was evaluated in reaction mixtures containing or not STS. Whereas the STS addition did not change MTA abundance in *C. albicans* culture. Conversely, the STS supplementation increased the MTA abundance by more than sixfold in Lcr35 culture. A significant increase in MTA abundance was also pointed out when STS was added to a co-culture between *C. albicans* and Lcr35. The low abundance of MTA was near to MS noise level in the control (culture medium supplemented with STS) confirming that the increase in MTA abundance cannot be due to its presence as an impurity in STS preparation.

### S-methyl thioacetate potentiates the anti-*Candid*a effect of Lcr35

**Figure 6:**
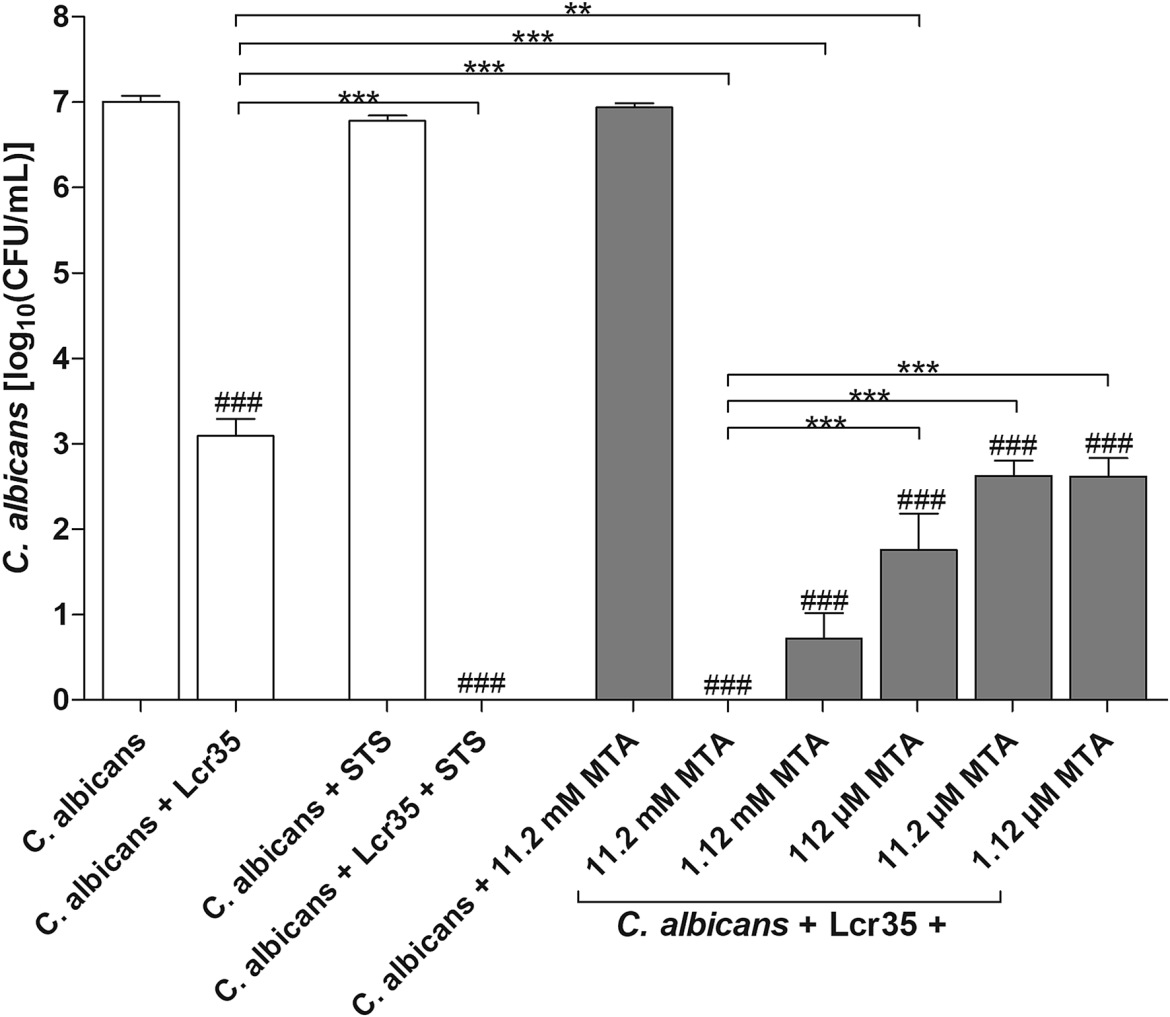
The anti-*Candida* ATCC 10231 effect of Lcr35 is potentiated in the presence of S-methyl thioacetate. Viability of *C. albicans* determined after 24 h of co-incubation with Lcr35 white bar, with Lcr35 and STS (1 g/l) white bar and Lcr35 with MTA grey bar. As control, *C. albicans* was incubated in MRS, MRS with 1 g/l STS and MRS with 11.2 mM MTA. N = 3, *p < 0.05, **p < 0.01, ***p < 0.001, significant difference between the condition and its control ^###^p < 0.001 (one-way ANOVA test and Bonferroni correction).

The potentiating effect of MTA was evaluated using a concentration range 11.2 mM to 1.12 µM (Fig. 6). *C. albicans* ATCC 10231 was susceptible to Lcr35 with MTA. In contrast, MTA alone had no antimicrobial effect against *C. albicans*. Concentration 11.2 mM of MTA induced the most significant anti-*Candida* effect of Lcr35, similarly to supplementation with STS (1 g/l). The anti-*Candida* effect of Lcr35 was not potentiated with concentrations of MTA lower than 11.2 µM.

## Discussion

The beneficial properties of microorganisms are usually evaluated with native culture strains. However, LBM are often administered as complex manufactured formulated products and positive impacts of the manufacturing process have been previously demonstrated in several studies^22–24^. In this study, we demonstrated that the vaginal formulation of *Lactobacillus rhamnosus* Lcr35, GYNOPHILUS, was able to kill *C. albicans* ATCC 10231 after 48 h, whereas the native strain alone only reduced the pathogen viability by 3 log_10_ (CFU/ml) over the same length of time^22^. This vaginal LBP is composed of the LBM Lcr35 and of the excipient sodium thiosulfate. We showed that this component potentiates the anti-*Candida albicans* activity of the 8 *Lactobacillus* strains tested in this work, suggesting a *Lactobacillus* genus impact of STS. In addition, an inhibitory effect of Lcr35 in the presence of STS was observed against a large panel of *Candida* spp. strains, evidencing a large range of inhibitory capacities of Lcr35 on *Candida* spp.

SEM observations showed *C. albicans* ATCC 10231 cells in close contact with Lcr35 cells after 48 h of co-culture (Fig. 3a). However, we demonstrated that physical interaction was not essential for Lcr35 anti-*Candida* activity. Moreover, cell free supernatant of Lcr35 inhibits *Candida* growth and this effect is potentiated in the presence of STS. These results suggest the presence of anti-microbial effectors in the extra-cellular medium of Lcr35, the production, release and/or stability and efficacy of which could be enhanced by STS. Some lactic acid bacteria, including *Lactobacilli* species, have anti-*Candida* activity, probably by direct inhibition, through competition for adhesion sites, biofilms formation, filamentation or interference on virulence genes^25^. Some underlying mechanisms involved the produ ction of exopolysaccharides or chitinase by *L rhamnosus* GG able to interfere with hyphal formation of *Candida*^26,27^. In this study, we looked for the loss of viability of *C. albicans* induced by Lcr35. The production of secondary metabolites (hydrogen peroxide or lactic acid) able to lyse fungal cells has been previously described^28,29^, but these hypotheses did not apply to Lcr35 since its antifungal activity was not modified using buffered and catalase-enriched media (data not shown).

Analysis of the Lcr35 cell free supernatant showed that the potential extra-cellular components involved in *C. albicans* ATCC 10231 growth inhibition were present in the fraction of less than 10 kDa. Among the small molecules secreted by *Lactobacilli*, some secondary metabolites have been reported like antimicrobial peptides or VOCs^30,31^. Since the investigation of antimicrobial peptides was unsuccessful (data not shown), we focused on the characterization of the VOCs produced by Lcr35 in the presence of STS. VOCs are typically small compounds with low molecular mass, odorous, high vapor pressure, low boiling point and a lipophilic character^30,32^. In addition, STS is composed of two sulfur atoms, and this element is known for its odoriferous properties when it is embedded in some molecules. It has been shown already that VOCs produced by the bacteria *Paraburkholderia graminis* were able to inhibit hyphal growth of the fungi *Rhizoctonia solani*^33^. Moreover, in the context of soil, prominent mediator molecules involved in keeping this complex ecosystem in balance, are inorganic and organic microbial volatile compounds (mVOCs)^34^. Among VOCs, sulfurous VOCs such as dimethyl di- and trisulfide have been previously shown to have an effect on growth of several plant pathogenic fungi^35–37^. In our study, a non-verbal sensory experiment showed that Lcr35, with or without STS, develops a particular and perceptible odor in the presence of *C. albicans*. GC-8O/MS analyses identified six odor-active compounds, including one sulfur compound that was identified as *S*-methyl thioacetate (MTA). The bacterial synthesis of this thioester can occur through two pathways, the alcoholysis of acyl-CoA via an acyl alcohol transferase or the chemical esterification of an organic acid with an alcohol^38^. Genes potentially involved in these two pathways, *i.e*. encoding putative acetyl-CoA acetyltransferase and thioesterases, respectively, as well as homologous of genes encoding a two-component system involved in the regulation of the biosynthesis of MTA in *Pseudomonas*^39^ are present in the genome of Lcr35 ; their analysis will contribute to characterizing MTA production in this bacterium. In our study, MTA when combined with Lcr35 presented an anti-*Candida* effect, suggesting that this thioester is an active metabolic intermediate. In addition, we showed that STS potentiates the anti-fungal action of several *Lactobacillus* species; it is thus likely that MTA fulfills a similar universal function. Indeed, MTA has already been reported as an antifungal VOC in bacterial soil^39^ but its mechanism of action remains to be elucidated.

In conclusion, ours results show that STS is an active excipient that contributes to the anti-*Candida* properties of the Lcr35 containing LBP GYNOPHILUS via an unknown mechanism involving MTA. Further postgenomic investigations of the interaction LBM—*Candida* will be necessary to elucidate the molecular mechanism driving the enhanced anti-*Candida* properties of Lcr35 in presence of STS. Nevertheless, our data demonstrate that STS could be widely used as a potentiator of LBM capacities and be included as excipient in LBP formulation to promote the beneficial effect.

## Materials and methods

### Fungal, bacterial strains and culture conditions

Stains used in this study are referred in Table 1. *Lactobacillus* spp*.* strains were grown (inoculated at 1.10^6^ CFU/ml) in Man, Rogosa, Sharpe (MRS) broth (bioMerieux, Marcy l’Etoile, France) at 37 °C for 48 h. *Candida spp.* strains were inoculated at 10^6^ CFU/ml in Sabouraud dextrose broth (bioMerieux, Marcy l’Etoile, France) and grown at 25 °C for 48 h^22^.

**Table 1:** Strains studied.

Taxonomic designation	Strains	References
*Candida albicans*	ATCC 10231	^40^
*C. albicans*	*1423*	Clinical sample from respiratory tract, this study
*C. albicans*	*6A*	Clinical sample from respiratory tract, this study
*C. albicans*	*BD2015*	Clinical sample from respiratory tract, this study
*C. albicans*	*BD2016*	Clinical sample from respiratory tract, this study
*C. albicans*	*F9*	Clinical sample from respiratory tract, this study
*C. albicans*	*PBp0020*	Clinical sample from respiratory tract, this study
*C. albicans*	*VL*	Clinical sample from respiratory tract, this study
*C. krusei*	*PBp0008*	Clinical sample from vaginal tract, this study
*C. tropicalis*	*PBp0018*	Clinical sample from vaginal tract, this study
*C. glabrata*	*PBp0016*	Clinical sample from vaginal tract, this study
*Lactobacillus casei*	CIP 103137	^41^
*Lactobacillus crispatus*	CIP 102990	^42^
*Lactobacillus gasseri*	CIP 102991	^43^
*Lactobacillus jensenii*	CIP 69.17	^44^
*Lactobacillus plantarum*	CIP 102021	^45^
*Lactobacillus reuteri*	CIP 101887	^46^
*Lactobacillus rhamnosus*	CIP A157	^47^
*Lactobacillus rhamnosus*	Lcr35	^48^
*Lactobacillus vaginalis*	CIP 105932	^49^

STS (PANREAC, Barcelona, Spain) was added at 1 g/l in the growth media i.e. either MRS or Sabouraud dextrose broth. S-Methyl thioacetate (MTA, CAS number 1534-08-3, Sigma-Aldrich, St-Quentin-Fallavier, France) was added at different concentration (11.2 mM to 1.12 µM by 1:10 dilutions) in the growth media of Lcr35 to determine the dose effect of anti-*Candida* potential.

For quantification of cultivable cells, the number of viable cells was determined by a plate count method on MRS agar (bioMerieux, Marcy l’Etoile, France) for detection of *Lactobacillus* spp. and Sabouraud agar for *Candida* strains. MRS plates were incubated at 37 °C for 72 h and Sabouraud petri dishes at 25 °C for 120 h^22,50^. The results were expressed as colony forming units per milliliter (CFU/ml) and the limit of detection was 10 CFU/ml.

### Anti-Candida effect

After 48 h of preculture, a mixed culture broth was made with 50% of *Lactobacilli* subculture containing or not STS (from 0.5 to 5 g/l) or MTA (11.2 mM to 1.12 µM) and 50% of *Candida* subculture. The cells were then grown for 24 h or 48 h at 37 °C. Controls included pure cultures of *Candida* spp. or *Lactobacillus* spp. with or without STS or MTA. The pH was monitored during the whole experiments.

Similar experiments were performed using cell culture inserts (TRANSWELL, Dutsher, Brumath, France). The membrane cell culture inserts, with 0.4 µm-pore-size polycarbonate membrane inserts, were placed in 12-well plates containing a final volume of 2 ml. Then, Lcr35 subculture with or without STS (1 g/l) was added into six-well cell culture plates and *C. albicans* ATCC 10231 subculture was added into the upper chambers and vice versa. The culture plates were incubated at 37 °C for 24 h. Controls were performed included pure cultures of *C. albicans* or Lcr35, with and without STS (1 g/l) and a co-culture without cell culture inserts. The viability of microorganisms was determined. Each assay was performed in triplicate.

The cell viability was tested using flow cytometry. The abundance of dead *C. albicans* cells was measured during co-incubation; one milliliter of the suspension was centrifuged (11,000×*g*, 10 min, at room temperature), and re-suspended in PBS (pH 7; Lonza, Levallois-Perret, France). Propidium Iodide (PI; Sigma-Aldrich, St-Quentin-Fallavier, France) was added (1 µg/ml) before incubation in the dark for 40 min at room temperature. One hundred microliters of each sample were seeded in 96 well plates^51^. The flow cytometry analysis was performed on a BD Accuri™ C6 Cytometer (BD Biosciences, Le Pont de Claix, France) and data were analyzed using the CFlow PLUS version 1.0 software (BD Biosciences). At least 2.10^5^ elements per sample were analyzed at a slow flow rate (14 µl/ml). Yeast and bacteria were discriminated in two different areas (P1 and P2). Two controls, one with live Lcr35 or *C. albicans*, and another with heat shock (100 °C, 15 min) killed Lcr35 or *C. albicans* were used to calibrate the gate.

### Inhibition of pathogens growth using cell free supernatant

The cell-free supernatants were collected after 48 h of *L. rhamnosus* Lcr35 subculture with or without STS and then centrifuged (10,000×*g*, 15 min) and filtered through a 0.2 µm filter (Millex; Merck, Darmstadt, Germany). The supernatants were diluted to obtain a proportion range of 10%, 30% and 50% in Sabouraud dextrose broth. *C. albicans* ATCC 10231 was inoculated at 10^6^ CFU/ml in medium containing the supernatant dilutions and incubated at 37 °C during 24 h. The *C. albicans* growth was followed by reading OD_600 nm_ every 15 min after shaking with automate Tecan microplate readers (TECAN, Genius, Männedorf, Switzerland). The controls were performed with a proportion range of MRS broth with 1 g/l STS and proportion range of sterile water.

Similar experiments were performed with cell free supernatants boiled (15 min at 100 °C) or treated with 0.2 mg/ml pronase E (Merck KGaA, Darmstadt, Allemagne.) and 0.2 mg/ml proteinase K 0.2 mg/ml (Sigma-Aldrich, St-Quentin-Fallavier, France) during 2 h at 37 °C. The enzymes were inactivated by thermal shock at 80 °C during 20 min.

The supernatant ultrafiltration was performed using vivacell100 (Sartorius, Dourdan, France) (20 min at 2000×*g*) using a 100 kDa 10 kDa and cut-off membrane. The residue was subsequently solubilized with an equivalent volume of H_2_O. The residual anti-*C. albicans* activities of each supernatant fraction was evaluated by reading OD_600 nm_ using proportion range of 50%. Untreated cell free supernatant was included in each experiment as control.

### Electron microscopy analysis

Samples were treated as previously described^52^. Details of the technical process were done into the supplemental materials and methods section.

### Sample preparation for Purge and trap gas chromatography–mass spectrometry analysis

The filtered microbial cultures were aliquoted in 4 ml screwed caps vials and stored at − 20 °C. Analyses were done on the culture media corresponding to each of the 7 treatments (*C. albicans*; MRS broth + STS; *C. albicans* + STS; Lcr35 + *C. albicans*; Lcr35; Lcr35 + STS; Lcr35 + *C. albicans* + STS) with three replicates. The vials were left to thaw overnight at 4 °C, the sample was homogenized by 5 s vortexing just before extraction.

### Assessment of odor dissimilarity by non-verbal sensory analysis

A panel of 25 untrained judges was recruited from the research teams of the University Clermont-Auvergne. To avoid altering their olfactory sense, the judges were asked the day of the session not to perfume themselves, not to consume anything after their breakfast except water and to avoid strong or predominant atmospheres (solvents, cigarettes, fermenters…). Three sessions (one per culture time studied: T = 0 h, T = 12 h and T = 24 h) of about 30 min each were organized at 10h45 am over 15 days. The analyses were carried out in a room equipped with individual boxes. Six to seven vials closed with screw caps containing the different culture media numbered using 3-digit numbers and randomized were placed on a sheet of paper (40 × 60 cm^2^) in front of each judge. The judges were instructed to smell the vials several times to compare them. After smelling them, they had to position them on the sheet of paper using the entire surface so that two vials were as closer as their odors were similar and were as distant as their odors appeared different. Once the positioning was complete, the vial numbers were recorded on the paper where the vials were placed. The x and y coordinate points of each vial were then recorded. For each napping session, a distance matrix was calculated from the vial coordinates: $\sqrt{{\left({x}_{2}-{x}_{1}\right)}^{2}+{\left({y}_{2}-{y}_{1}\right)}^{2}}$, x and y representing the coordinates of the points.

### Volatile compounds analysis by dynamic headspace-gas chromatography-mass spectrometry (DH-GC–MS) and Olfactometry

DH–GC–MS analyses were performed on three replicates of seven conditions corresponding to culture media with or without STS inoculated or not with *C. albicans*, Lcr35 or both, as described in supplemental materials and methods section.

The odorous compounds of 4 selected conditions (Lcr35; Lcr35 with STS; Lcr35 and *C. albicans;* Lcr35 with STS and *C. albicans*,) were analyzed by eight-way olfactometry coupled to gas chromatrography—mass spectrometry (DH–GC–MS/8O)^53,54^. The analyses consisted in the simultaneous acquisition of the GC–MS signal of the sample in one side and the GC-8O signal consisting in the odor profile detected by the panel of eight judge in the other side. Details of the procedure were mentioned on the supplemental materials and methods section.

### Statistical analysis

Statistical analyses were performed using a one-way ANOVA test followed by Bonferroni correction for growth experiments. Statistical analyses were carried out using GraphPad Prism 5 software (www.graphpad.com/prism) (*p < 0.05; **p < 0.01; ***p < 0.001).

Data treatment of sensory analyses were processed and analyzed using STATISTICA version 13 software (Statsoft, Maisons-Alfort, France). For assessment of odor dissimilarity, a panel distance matrix was constructed by averaging the individual distance matrices of judges (n = 25). The resulting distance matrix was processed by Multi-Dimensional Scaling (MDS). The number of dimension of the model was set based on 1/ Stress value, 2/ Alienation value and 3/ Shepard diagram following the recommendation of Ding, 2018.

For the determination of distinct odor-active compounds by DH-GC-MS/8O, a Kruskal–Wallis nonparametric test (p < 0.05) was processed on the intensity given by each judge for each odor zone. The relevant odor-active compounds related to each significant odor zones were considered as distinct markers of the four cultures.

For the screening of sulfur compounds as markers of culture discrimination, one-way ANOVA (model: peak area = culture, p < 0.05) were performed on triplicates of DH-GC–MS peak areas. Tukey multiple mean comparison tests (p < 0.05) were processed on significant sulfur compounds to investigate the significance of inter-culture differences.

Statistical analysis of gas chromatography–mass spectrometry were performed using Statistica Software. One-way ANOVAs were performed on peak areas of the specific ion (m/z) of each volatile compound to identify significant effects of the treatments (abundance of compound specific ion: treatment, p < 0.05). Newman-Keuls multiple comparison tests were performed to determine significant differences between treatments (p < 0.05).

## Supplementary information

Supplementary Information.
