# Supplementary material for: Identification of sulfur components enhancing the anti-Candida effect of Lactobacillus rhamnosus Lcr35
Source: Sci Rep. 2020 Oct 13;10:17074. doi: 10.1038/s41598-020-74027-7 (PMC7553951; doi:10.1038/s41598-020-74027-7)
Supplement: Supplementary file 1 — Supplementary Information. [file 41598_2020_74027_MOESM1_ESM.docx]

**Identification of sulfur components enhancing the anti-*Candida* effect of *Lactobacillus rhamnosus* Lcr35.**

**Supplementary information**

Caroline Dausset^a,b,c,#^, Sylvie Miquel^c^, Stéphanie Bornes^d^, Nathalie Kondjoyan^e^, Magaly Angenieux^e^, Laurence Nakusi^c^, Philippe Veisseire^d^, Elina Alaterre^a*^, Luis G. Bermúdez-Humarán^b^, Philippe Langella^b^, Erwan Engel^e^, Christiane Forestier^c&^ and Adrien Nivoliez^a&^

*^a^ Research and development department, BIOSE, 15000, Aurillac, France;*

*^b^ Micalis Institute, INRA, AgroParisTech, Université Paris-Saclay, 78350, Jouy-en-Josas, France;*

*^c^ Université Clermont Auvergne, CNRS LMGE, F-63000, Clermont-Ferrand, France*

*^d^ Université Clermont Auvergne, INRA, VetAgro Sup, UMRF, F-15000, Aurillac, France*

*^e^ INRA, UR370 QuaPA, Microcontaminants, Aroma & Separation Science group (MASS), F-63123 Saint-Genès-Champanelle, France*

**Present address: HORIBA ABX SAS, Parc Euromédecine, Rue du Caducée, BP 7290, 34184 Montpellier Cedex 4, France*

*^&^ Equal contributors*

*^#^ Address correspondence to Caroline Dausset; BIOSE, 24 avenue Georges Pompidou, 15000 Aurillac; c.dausset@biose.com; +334 71 46 80 00.*

**Supplemental materials and methods**

## Electron microscopy analysis

For scanning electron microscopy observation, the cells were washed in rinsing buffer and fixed for 48 hours at 4°C in 0.2 M sodium cacodylate buffer, pH 7.4 containing 0.05 % red ruthenium and 1.6% glutaraldehyde. Microorganisms were then washed 10 minutes in sodium cacodylate buffer (0.2 M, pH 7.4) with 0.05 % of red ruthenium. They were post-fixed 1 hour with 1% osmium tetroxide and 0.05 % red ruthenium in 0.2 M sodium cacodylate buffer (pH 7.4) and washed 20 minutes in distilled water. Dehydration by graded ethanol were performed from 25°C to 100°C (10 minutes each) to finish in hexamethyldisilazane (HMDS) 10 minutes. Microorganisms were deposited on Thermanox slides and dried at room temperature. Samples were then mounted on stubs using adhesive carbon tabs and sputter-coated with gold-palladium (JFC-1300, JEOL, Japan). Morphological analysis was carried out using a scanning electron microscope JSM-6060LV (Jeol, Japan) at 5kV in high-vacuum mode.

For transmission electron microscopy, microorganisms were washed three times in sodium cacodylate buffer (0.2 M, pH 7.4), post-fixed 1 hour with 1% osmium tetroxide in 0.2 M sodium cacodylate buffer (pH 7.4) and washed three times (10 minutes) in sodium cacodylate buffer (0.2 M, pH 7.4). Specimens were then dehydrated in a graded ethanol and acetone. Subsequently, they were infiltrated with acetone and EPON resin mixture (2:1) for 1 h, with acetone and EPON resin mixture (1:1) for 1 h and with acetone and EPON resin mixture (1:2) for 1 h. Specimens were embedded in resin overnight at room temperature and cured 2 days in a 60°C oven. Thin sections (70 nm) were obtained using a UC6 ultramicrotome (Leica, Germany) and stained with uranyl acetate and Pb citrate. Microorganisms sections were observed with a transmission electron microscope (Hitachi H-7650) at 80 kV acceleration voltage. Micrographs were made using a Hamamatsu AMT 40 camera placed in a side position.

## Volatile compounds analysis by dynamic headspace-gas chromatography-mass spectrometry (DH-GC-MS)

The filtered microbial cultures were aliquoted in 4 ml screwed caps vials and stored at −20 °C until further analysis. The vials were left to thaw overnight at 4°C and the samples were homogenized by 5 second vortexing just before extraction. The volatile compounds were extracted by “purge and trap” dynamic headspace (Tekmar, Cincinnati, OH 45234, USA). To limit foam overflow during sample purge, a headspace sampler system made of two cartridges (E70100-25ml, EST analytical, 503 Commercial Drive Fairfield, Ohio 45014) linked by a stainless-steel tube (12.7 mm diameter, 1 mm thickness, 32 cm length) has been used. Two milliliters of vortexed samples were introduced in the headspace sampler system. The extraction parameters were as follows: purge 30 min at room temperature by a 3.6 l/h helium flow, Tenax trap (180 mm × $\frac{1}{8}$” id, packed with TENAX TA 60/80 mesh adsorbent, Supelco Bellefonte PA 16823, USA), dry-purge for 5 min, desorb for 10 min at 215 °C on a cryotrap at -150 °C in the chromatograph injection port. Separation was performed on a 6890A gas chromatograph hyphenated with a 5973-mass selective detector (Agilent Technologies, Santa Clara, CA). The extract was injected by thermodesorption of the cryotrap for 2 min at 220 °C on a DB5-MS UI column, (60 m × 0.32 mm id × 1 μm, Agilent Technologies, Les Ulis, France). The oven temperature program was as follows: 40 °C for 5 min, then increasing at a rate of 4 °C min−1 up to 230 °C then held for 10 min. Volatile compounds were tentatively identified on the basis of mass spectra and retention indices (RI), by comparison with the NIST/EPA/NIH mass spectral library (NIST14) and with our internal LRI database 38 ^1^. For sulfur compound search, a systematic screening has been done on the chromatograms of the seven conditions studied. Sulfur compounds were semi-quantified by measuring the peak areas of their specific ions with the extracted ion chromatogram mode of the spectrometer software (MSD Chemstation E. 02. 02. 1431, Copyright © 1989-2011 Agilent Technologies, Inc., Santa Clara, CA).

## Odor-active compound profiling by DH-GC-MS/Olfactometry

The gas chromatography – mass spectrometry data acquisition was performed as in previous section except for the extraction conditions where two milliliters of the vortexed samples were introduced in the two cartridge headspace sampler system, purged 45 min on a Tenax trap (180 mm × ¼” id). The sample was then drained and replaced by 1 ml of the same media and purged once more for 15 min in order to enrich the extract with low molecular weight compounds. The separation has been performed on a 6890A gas chromatograph hyphenated with a HP 4440 Chemical Sensor spectrometer (Hewlett-Packard Enterprise, San José, California, USA), with a RTX5 column, (60 m × 0.53 mm id × 1.5 μm (Restek, 7 avenue du Général de Gaulle, 91090 Lisses, France. For the olfactometric analyses, the 8 sniffers were non-smokers with no known health disorders, recruited in the INRA ARA research centre. They were selected for their ability to detect and describe odors by GC-Olfactometry. They did not know the nature of the samples. They were asked the day of the session not to perfume themselves, not to consume anything after their breakfast except water and avoid strong or predominant atmospheres. To measure the intensity of the odors, they were asked to rate the odor with a five-level scale (1, very weak; 2, weak; 3, moderate; 4, strong, and 5, very strong), and in the same time, they had to qualify it by giving a descriptor of the odor. Olfactometric analyses lasted 35 min. Data were acquired and processed with AcquiSniff Software^2^, giving the reconstructed aromagram based on the total olfactive signal, ${TOS}_{Int x Det}$ from the 8 judges expressed as a product of average score intensity and detection frequency in order to locate the odor-active zones commonly perceived.^3^ Finally, the identification of the odor-active compounds of each odor-zone was realized by matching data from their linear retention indices (LRI), mass spectra, injection of relevant standard compounds and odor databases (NIST14, internal LRI database^1^, The Good Scent Company (tgsc) TM, Copyright © 1980-2018, unpublished internal odor database) with the odor-zone descriptions.

1. Kondjoyan, N. & Berdagué, J.-L. A compilation of relative retention indices for the analysis of volatile compounds. *Edition Journal of Dairy Science* (1996).

2. Berdagué, J. L. & Tournayre, P. The ‘ Video-Sniff’ method, a new approach for the ‘ vocabulary-intensity-duration’ study of ‘ elementary odours’ perceived by gas-chromatography-olfaction. in *Flavour Research at the Dawn of the twenty-first century* 514–519 (2003).

3. Berdagué, J. L., Tournayre, P. & Cambou, S. Novel multi-gas chromatography-olfactometry device and software for the identification of odour-active compounds. *J. Chromatogr. A* **1146**, 85–92 (2007).

**Supplementary data**


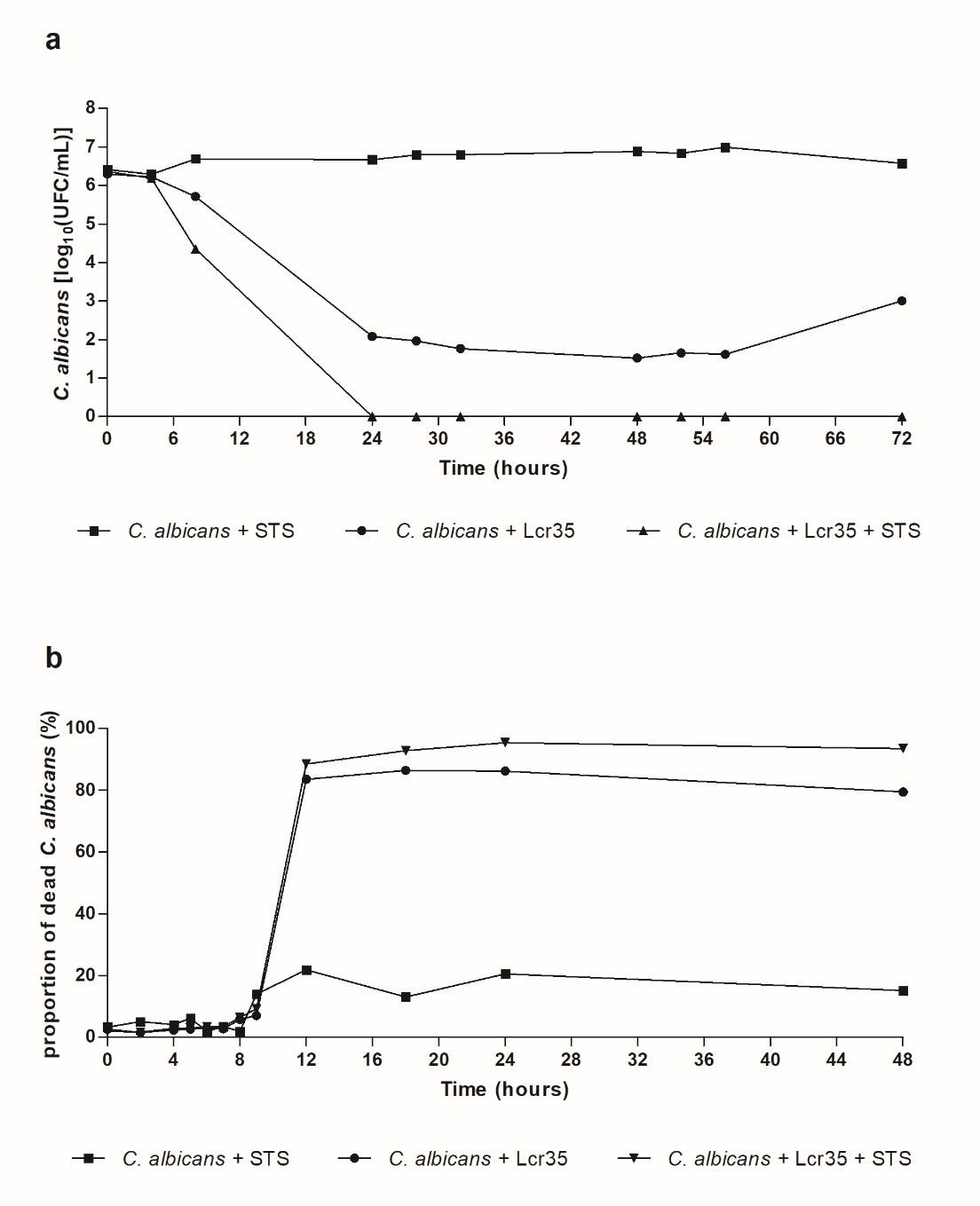


**S1: Viability of *C. albicans* during co-incubation with Lcr35 with and without STS.**

Viability of *C. albicans,* by plate counting (a) and cytometry (b) determined with propidium iodide treatment, at strain contacting (T0), after 24 h (T24) and 48 h (T48) of co-incubation with Lcr35.


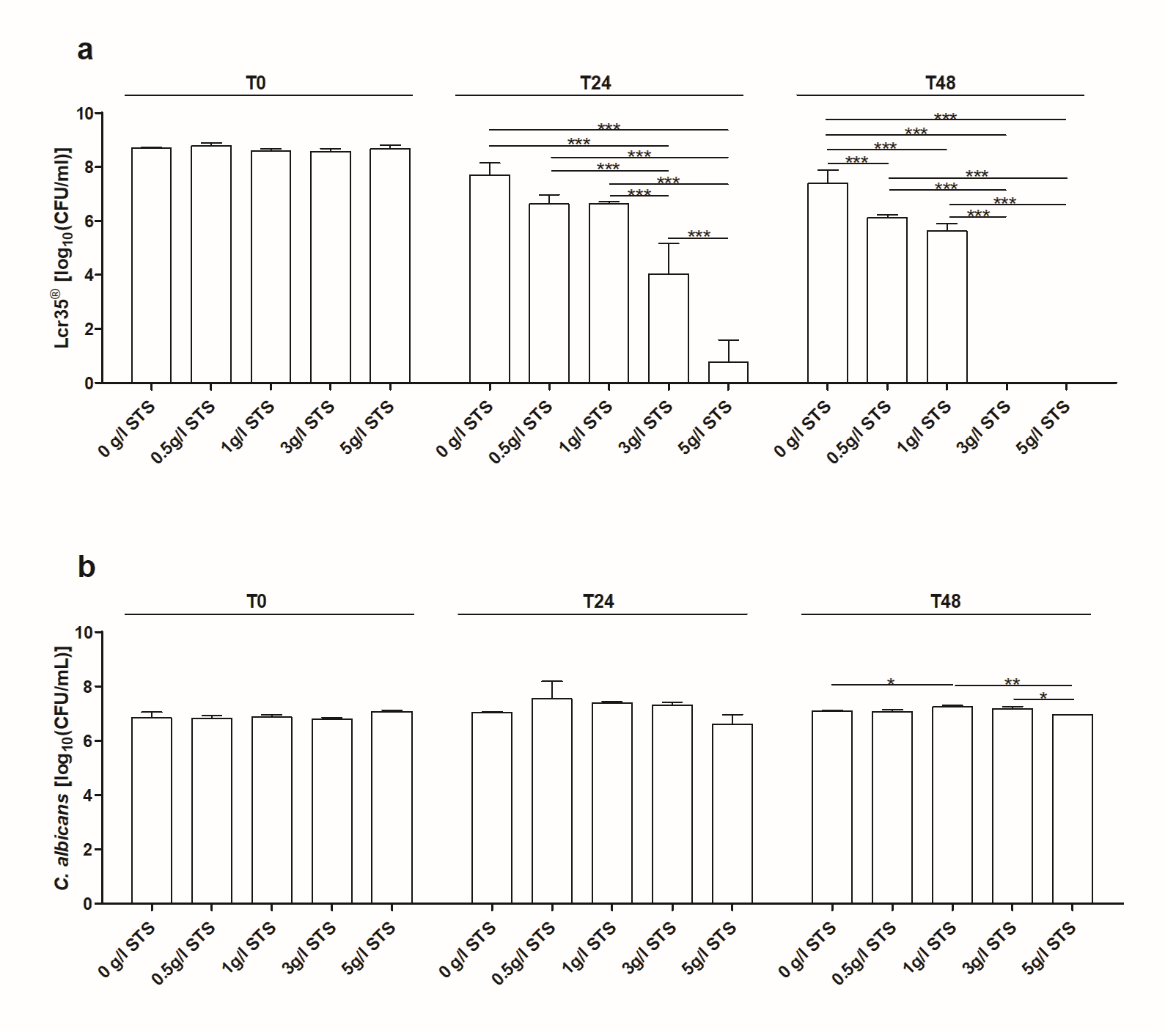


S2: Effect of STS on the viability of Lcr35 and *C. albicans*

Viability of Lcr35 (a) and *C. albicans* (b) was determined when strain contact occurred (T0) and after 24 h (T24) and 48 h (T48) of presence with concentration range of STS (0 g/l to 5 g/l). N=3, * p < 0.05, ** p < 0.01, *** p < 0.001 (one-way ANOVA test and Bonferroni correction).


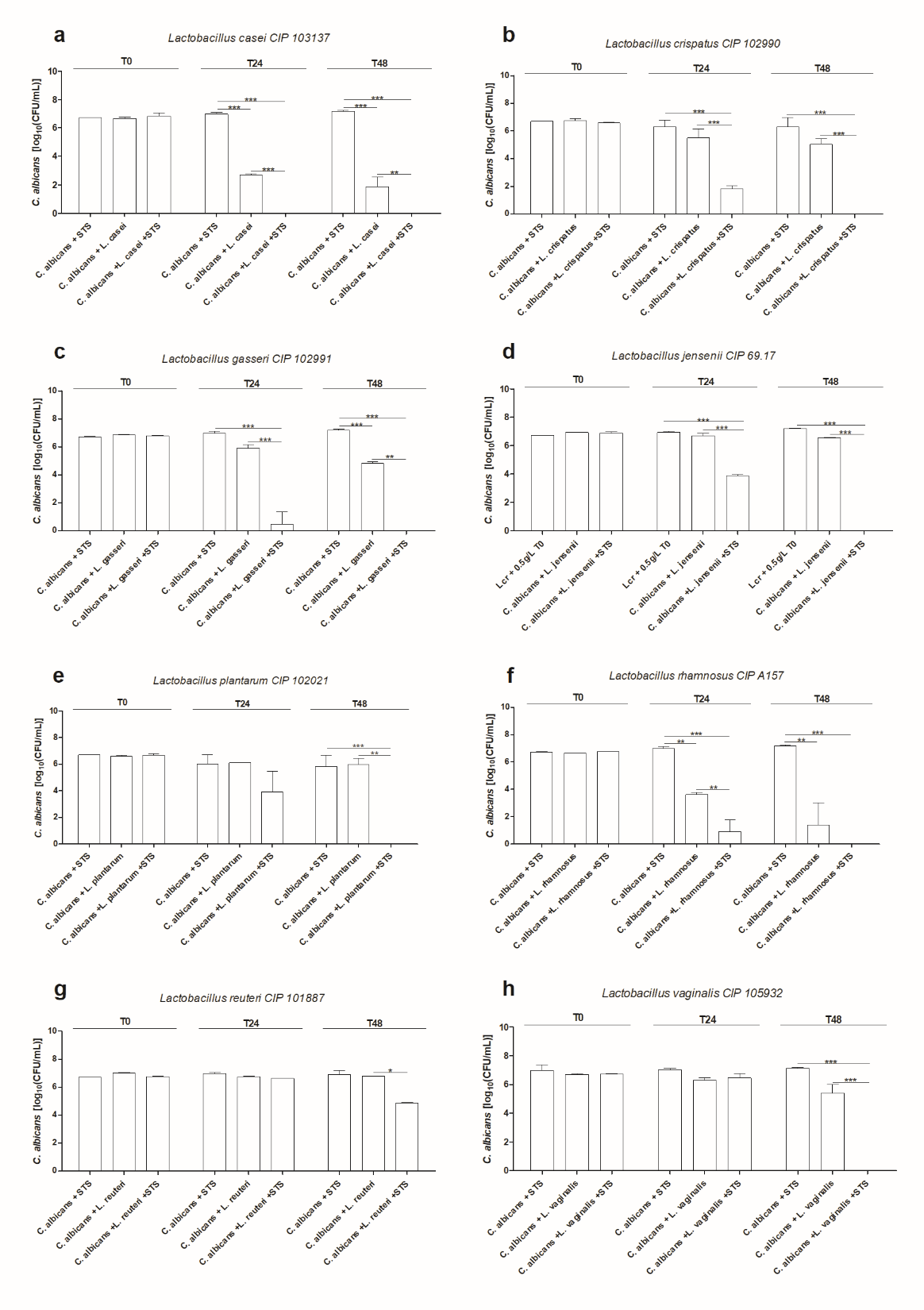


**S3: Anti-*Candida* activity of *Lactobacillus* spp.**

Viability of *C. albicans* determined at strain contacting (T0), after 24 h (T24) and 48 h (T48) of co-incubation with *Lactobacillus casei* CIP 103137 (a), *Lactobacillus crispatus* CIP 102990 (b), *Lactobacillus gasseri* CIP 102991 (c), *Lactobacillus jensenii* CIP 69.17 (d), *Lactobacillus plantarum* CIP 102021 (e), *Lactobacillus rhamnosus* CIP A157 (f), *Lactobacillus reuteri* CIP 101887 (g), *Lactobacillus vaginalis* CIP 105932 (h), with or without 1 g/l of STS. *C. albicans* was incubated with 1g/l STS as control. N=3, * p < 0.05, ** p < 0.01, *** p < 0.001 (one-way ANOVA test and Bonferroni correction).


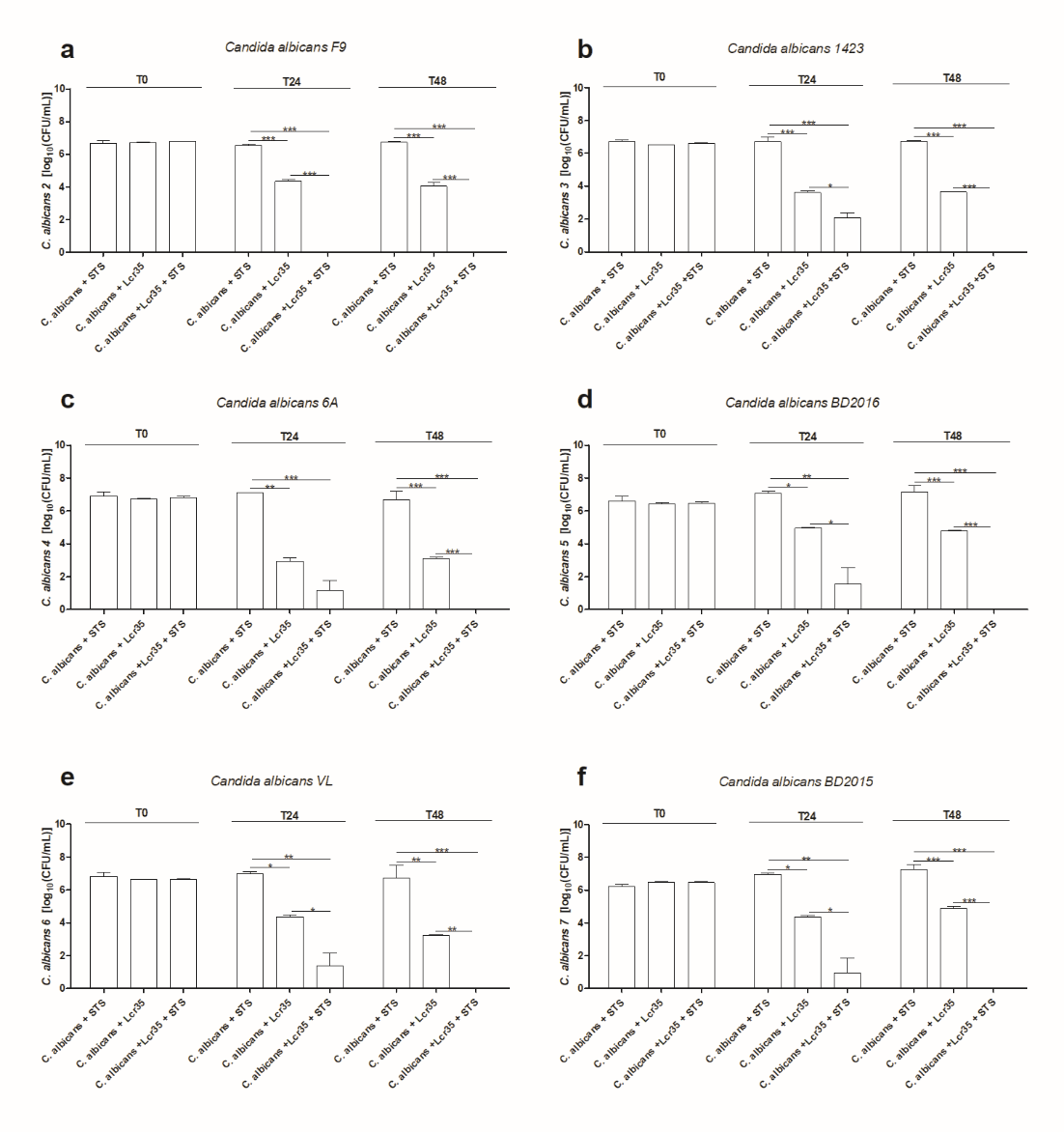


**S4: Capacity of Lcr35 combined with STS to inhibit several clinical strains of *Candida* spp.**

Viability of clinical strains of *C. albicans* (F9, 1423, 6A, BD2015, VL, D2015) at strain contacting (T0), after 24 h (T24) and 48 h (T48) co-incubation with Lcr35 with or without 1 g/l of STS. As controls, *Candida* spp. were incubated without Lcr35 and with 1 g/l STS. N=3, * p < 0.05, ** p < 0.01, *** p < 0.001 (one-way ANOVA test and Bonferroni correction).

**S5: List of identified odor-active compounds from the average profile of the DH-GC-MS/8O analyses of *C. albicans* +Lcr35®, *C. albicans* + Lcr35® +STS, Lcr35®, Lcr35^®^ + STS cultures and a short description of their perceived odor by the panel.**

| **Peak number^1^** | **Retention Time (s)^2^** | **LRI^3^** | **Chemical name^4^** | **CAS number** | **Odor-zone^5^** | **Kruskal-Wallis test^6^** |
| --- | --- | --- | --- | --- | --- | --- |
| 1 | 296 | nd | Acetaldehyde^a^ | 75-07-0 | Alcoholic |  |
| 2 | 358 | nd | Ethanol^a^ and acetone^a^ | 64-17-5; 67-64-1 | Butter, floral |  |
| 3 | 473 | 550 | Propanal, 2-methyl-^a^ | 78-84-2 | Cardboard, ammoniacal |  |
| 4 | 530 | 600 | 2,3-butanedione^a^ | 431-03-8 | Butter, milk |  |
| 5 | 690 | 657 | Butanal, 3-methyl-^a^ | 590-86-3 | Gratin, chemical |  |
| 6 | 916 | 732 | Butanol, 3-methyl-^a^ | 123-51-3 | Roquefort, ammoniacal |  |
| 7 | 987 | 756 | 2-pentanone, 3-methyl-^b^ | 565-61-7 | Strawberry, floral |  |
| 8 | 1039 | 765; 769 | Butanoic acid^a^ and 1-pentanol^a^ | 107-92-6; 71-41-0 | Smoke, solvent, vomit |  |
| 9 | 1110 | 800; 811 | Hexanal^a^ and acetic acid, butyl ester^b^ | 66-25-1; 123-86-4 | Fruity, fresh grass |  |
| 10 | 1202 |  | Unknown 1 |  | Butter, cheese |  |
| 11 | 1263 | 852 | Butanoic acid, 3-methyl-, ethyl ester^a^ | 108-64-5 | Red fruit, hot rubber | * |
| 12 | 1340 | 875 | 1-hexanol | 111-27-3 | Rubber, hay | * |
| 13 | 1433 | 914 | Propanal, 3-methylthio-^a^ | 3268-49-3 | Baked potato | * |
| 14 | 1448 | 910 | Furan, 2-acetyl-^b^ and pyrazine, 2,5-dimethyl-^a^ and pyrazine, ethyl-^a^ | 1192-62-7; 123-32-0; 13925-00-3 | Solvent, ether, peanut | * |
| 15 | 1485 | 935 | Unknown 2 | 15726-15-5 | Lemon, orange |  |
| 16 | 1626 | 987 | 1-octen-3-one^c^ and/or 1-octen-3-ol^c^ | 4312-99-6; 3391-86-4 | Mushroom |  |
| 17 | 1642 | 994 | 1-octen-3-one^c^ and/or 1-octen-3-ol^c^ and trisulfide, dimethyl-^c^ | 4312-99-6; 3391-86-4; 3658-80-8 | Garlic, mushroom |  |
| 18 | 1691 | 1007 | Octanal^a^ | 124-13-0 | Grapefruit | * |
| 19 | 1757 | 1041 | Pyrazine, 2-methyl-6-vinyl-^b^ | 13925-09-2 | Gratin, burnt |  |
| 20 | 1827 | 1069 | 1-octanol^a^ | 111-87-5 | Soap, rose |  |
| 21 | 1891 | 1093 | 1-nonen-3-one^c^ and/or 1-nonen-3-ol^c^ | 24415-26-7; 21964-44-3 | Mushroom | * |
| 22 | 1920 | 1108 | Nonanal^a^ | 124-19-6 | Coffee, plastic, grass |  |
| 23 | 1938 | 1115 | Unknown 3 |  | Roasted peanut, animal |  |
| 24 | 2067 | 1172 | 1-nonanol^a^ and gamma-heptalactone^b^ | 143-08-8; 105-21-5 | Rose, bleach, soap |  |

The odor-active compounds of each odor-zone have been identified by DH-GC-MS/Olfactometry and DH-GC-MS on the same four culture media (Lcr35, *C. albicans* + Lcr35, Lcr35 + STS and *C. albicans* + Lcr35 + STS) and the matching of the odor described by the panelists with their odor according to literature data.

1: Peak number as given in the aromagram (Fig. 5); 2: Aromagram retention time; 3: Linear Retention Indices (LRI) calculated for the DB5-MS UI capillary column of the DH-GC-MS/8O system; 4: Proposed compounds. The reliability of their identification is: a: mass spectrum, retention index and odor identical to those of a co-injected standard - b: mass spectrum, retention index and odor in agreement with literature data - c: proposed compounds according to previous published data when they cannot be found in the DH-GC-MS/8O or DH-GC-MS signals. Some odor-zones can have 2 or 3 proposed co-eluted compounds which could be implicated in the global perceived odor; 5: Major descriptors given by the judges; 6: Significance of the Kruskal-Wallis (p<0.05), the significant compounds are pointed with a dark arrow in figure 5B.
